# An unusual case of pilonidal p16 positive squamous cell carcinoma—a case report

**DOI:** 10.1093/jscr/rjae076

**Published:** 2024-02-21

**Authors:** Hershil Khatri, Tzu-Yi Chuang, Daniel Swan

**Affiliations:** Department of General Surgery, Ipswich Hospital, Ipswich, QLD 4305, Australia; Department of General Surgery, Ipswich Hospital, Ipswich, QLD 4305, Australia; Department of General Surgery, Ipswich Hospital, Ipswich, QLD 4305, Australia

**Keywords:** case report, pilonidal, p16, squamous cell carcinoma

## Abstract

Basaloid squamous cell carcinoma (BSCC) is a rare and aggressive variant of squamous cell carcinoma. It is commonly seen in the oropharynx and strongly associated with p16-expressivity and high-risk human papilloma virus (HPV). We report the first case of primary cutaneous p16-positive BSCC in an elderly woman, with a background of chronic inverse psoriasis of the natal cleft. P16-expressivity is a common surrogate marker for oncogenic HPV16, routinely tested for oropharyngeal/anogenital squamous cell carcinoma. This is not routinely done for primary cutaneous disease. Pilonidal disease is uncommon in the elderly population, and malignant transformation is rarer still. Surgical resection is considered the mainstay of treatment for primary cutaneous BSCC, however due to this patient’s broad distribution of cutaneous field change and p16-expressivity, she was effectively treated with primary radiotherapy. This is a unique case of malignant transformation of pilonidal disease in an atypical demographic, with a rare/aggressive disease variant.

## Introduction

Squamous cell carcinoma (SCC) arising from chronically infected wounds (Marjolin Ulcer) is a well-known pathology, arising from chronic inflammation and aberrant cellular regeneration. Basaloid squamous cell carcinoma (BSCC) however is an aggressive variant of SCC, found most often the oropharynx [1, 2]. It is associated with human papilloma virus (HPV) infection, and the tumorigenesis pathway is well substantiated. Oncogenic variants of HPV (oncogenes E6 and E7 in particular) inhibit tumor suppressors P53 and Rb, which facilitates unregulated cellular proliferation and tumorigenesis [2]. HPV-16 in particular is commonly found in anogenital/oropharyngeal BSCC [3]. HPV-related p16-positive cutaneous disease however is a rare clinical entity [4]; to date, there have been eight reported cases of cutaneous p16-positive BSCC [3, 5–7]. We present a unique case of p16-positive BSCC of the natal cleft which presents a unique clinical dilemma regarding the roles of surgery, chemoradiotherapy, and immunotherapy in a context other than anogenital/oropharyngeal disease.

## Case report

An 80-year-old woman was referred to the surgical outpatient department for management of a chronic superficial sacral wound, present for ~2 years. Her presenting complaint was that of worsening sacral pain, and discomfort from local skin irritation. She had no prior history of pilonidal disease. There was no history of purulent discharge from the region. She denied any gastrointestinal symptoms including altered bowel habit, per-rectal bleeding, or rectal discharge.

The patient was a known actinopath, with a history of previous cutaneous non-melanoma skin cancers of the chest and ankle; she was previously known to a dermatologist for inverse psoriasis affecting her natal cleft. The patient had a coccygectomy 20 years prior, for a symptomatic benign bony protruberance. She had known ischemic heart disease with prior coronary artery bypass graft, hypertension, dyslipidemia, mild asthma, as well as an emergency splenectomy 26 years ago in the context of domestic violence. She was a remote ex-smoker. She was an active and independent individual and did not lead a particularly sedentary lifestyle. Her regular medications included aspirin, rosuvastatin, and valsartan. Topical corticosteroids were prescribed on an as-needed basis for flares of her inverse psoriasis.

**Figure 1:**
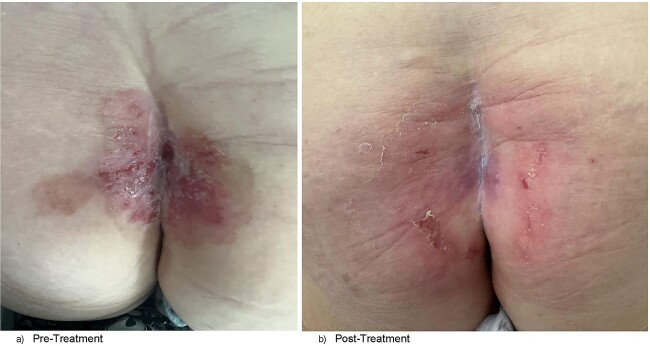
(a) Clinical photograph of patient’s natal cleft demonstrating broad region of excoriation with plaques, central sinus at superior aspect of the cleft, and prior coccygectomy scar in the left paramedian position. (b) Clinical photograph of patient’s natal cleft post-radiotherapy showing significant reduction of skin excoriation and plaque sizes and obliteration of the sinus tract.

Clinically, the patient was systemically well. She was not hirsute. Her prior coccygectomy scar appeared slightly indurated, surrounded by a broad region of excoriated skin and plaques at the upper natal cleft, and focal areas of ulceration (Fig. 1a). There was the appearance of a small sinus tract superiorly, but no obvious pits. There was mild serous discharge from regions of skin breakdown. It was >5 cm from the anal verge. There was no exophytic mass lesion, nor was there a clinically appreciable abscess cavity underlying. There was no palpable inguinal lymphadenopathy.

### Investigations

Two punch biopsies were taken in the outpatient setting on either side of the natal cleft. Full pathology report is attached in Supplementary Appendix A.

- right natal cleft, punch biopsy: BSCC arising from an intraepidermal carcinoma. P16 is positive.

- left natal cleft, punch biopsy: BSCC in situ which is also P16 positive. No invasive carcinoma is seen.

**Figure 2:**
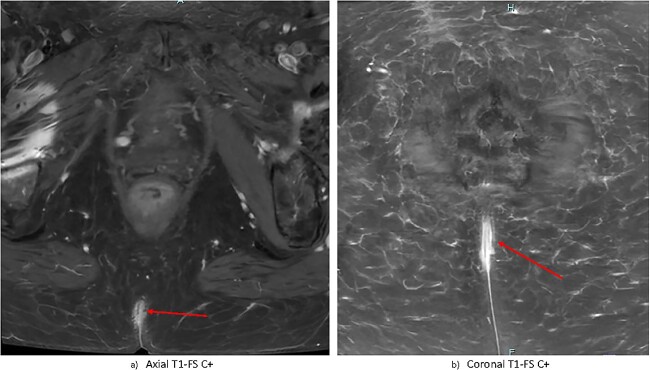
Gadolinium-contrast enhanced MRI images at time of diagnosis showing small-volume, superficial disease in (a) axial and (b) coronal planes. There is evidence of nonspecific superficial skin enhancement (arrows) but no evidence of significantly infiltrative disease or nodal metastases.

Contrast-enhanced magnetic resonance imaging (MRI) of the pelvis demonstrated nonspecific, irregular enhancement in the natal cleft measuring 6 × 13 × 28 mm, with no other visible mass lesion (Fig. 2). There was no visible invasion of underlying structures. There was no notable sinus tract demonstrated; however there was a small subcutaneous cystic focus noted 5 mm to the left of the superior margin of the natal cleft. There were no bony or regional nodal metastases. Full report in Appendix B.

Staging computed tomography (CT) scan of the chest, abdomen, and pelvis did not demonstrate any metastatic disease.

### Differential diagnoses

Prior to obtaining biopsy results, the differentials considered were varied. They included uncomplicated flare of known psoriatic condition, atypical pilonidal disease, and intertrigo. Malignancy such as a Marjolin ulcer and extramammary Paget’s disease required exclusion in the context of chronic inflammation and worsening sacral pain.

Pilonidal disease was considered as a differential based on the anatomical distribution of the lesion; however it would be unusual given the patient’s age as well as not having any regional hair growth.

### Management

This patient’s case was discussed at the colorectal multi-disciplinary team meeting. Consensus opinion was that surgery would not be ideal given the broad region that would require resection and likely poor wound healing. She was referred to the radiation oncology for definitive radiotherapy. She was reviewed in the surgical clinic 6 weeks post-definitive radiotherapy, at the time of which there was complete disease regression, and the patient no longer experienced any pre-treatment symptoms (Fig. 1b).

## Discussion

### Pilonidal disease

Pilonidal disease is common, with a prevalence of 0.026% [8]; common risk factors include male gender, obesity, sedentary lifestyles, excessive body hair, and excessive sweating [9]. In this patient, chronic inflammation from inverse psoriasis likely contributed to development of pilonidal disease despite not being the typical demographic. Furthermore, malignant transformation of pilonidal disease is rare [10].

Primary cutaneous p16-positive BSCC is a rare and aggressive malignancy. To date there have been eight cases reported in the literature, for which each patient had either nodal or distant metastases at time of diagnosis [3, 5–7]. This is the first reported case of p16-positive BSCC of the natal cleft. While not specifically tested for this patient, prior studies have shown a significant association between p16 expressivity and high-risk HPV (HR-HPV) oncogene positivity for primary cutaneous BSCC, and is considered to be a surrogate marker thereof [11]. Particularly in the context of oropharyngeal BSCC, p16-positivity confers a better prognosis than p16-negative disease, attributed to the molecular tumorigenesis secondary to HR-HPV strains [12]. Generally, surgical resection is the mainstay of treatment for cutaneous SCC. While not routinely screened for with primary cutaneous BSCC, p16-expressivity may help facilitate prognostication, as well as help guide adjuvant chemoradiotherapy as is in the case of oropharyngeal p16-positive BSCC [1]. Given the rarity of this condition, and paucity of literature available, further studies are certainly indicated to further delineate the chemosensitivity and radiosensitivity of primary cutaneous p16-positive BSCC as is seen in primary oropharyngeal disease.

### Learning points

1) Cutaneous p16-positive BSCC is a rare clinical entity. There are no previous reports of cutaneous p16-positive BSCC of the natal cleft.
2) P16 immunohistochemistry staining of BSCC is beneficial both from the perspective of available treatment options for patients, as well as for prognostication.
3) Malignant transformation of pilonidal disease is rare.
4) Clinicians should have a high index of suspicion for malignant transformation when investigating/managing chronic wounds.

## Supplementary Material

Appendices_rjae076

## Data Availability

Data sharing is not applicable to this article as no new data were created or analyzed in this study.
