# Supplementary material for: An unusual case of pilonidal p16 positive squamous cell carcinoma—a case report
Source: J Surg Case Rep. 2024 Feb 21;2024(2):rjae076. doi: 10.1093/jscr/rjae076 (PMC10881292; doi:10.1093/jscr/rjae076)
Supplement: Appendices_rjae076 [file appendices_rjae076.docx]

Appendix A: Pathology Report

**MACROSCOPY:**

1. Natal cleft R: The specimen consists of a punch of skin measuring 4mm in diameter and 7mm in depth. Bisected, all in, block 1. A PAS stain has been requested for this specimen.

2. Natal cleft L: The specimen consists of a punch of skin measuring 4mm in diameter and 6mm in depth. Bisected, all in, block 2. A PAS stain has been requested for this specimen.

**MICROSCOPY:**

*Natal Cleft R:*

The sections of punch biopsy show MODERATELY DIFFERENTIATED BASALOID SQUAMOUS CELL CARCINOMA. The carcinoma extends into the deep reticular dermis. There is no evidence of lymphovascular or perineural invasion. Surface ulceration and inflammatory granulation tissue are also identified.

The maximum tumour thickness is 2.5mm.

Clearance from the edge of the biopsy:

- Peripheral: INVOLVED

- Deep: 3mm

*Natal Cleft L:*

The sections of the punch biopsy show A HYPERTROPHIC AND BASALOID INTRAEPIDERMAL SQUAMOUS CELL CARCINOMA. There is no dermal invasion.

Clearance from the edge of the biopsy:

- Peripheral: INVOLVED

- Deep: 3mm

**CONCLUSION:**

*Natal Cleft Right, Punch Biopsy:*

- MODERATELY DIFFERENTIATED BASALOID SQUAMOUS CELL CARCINOMA (SCC)

- IMMUNOHISTOCHEMISTRY MARKERS ARE PENDING

*Natal Cleft Left, Punch Biopsy:*

- HYPERTROPHIC AND BASALOID INTRAEPIDERMAL SQUAMOUS CELL CARCINOMA (13)

- IMMUNOHISTOCHEMISTRY MARKERS PENDING

***SUPPLEMENTARY REPORT***

**MICROSCOPY:**

1. Synaptophysin and CD56 are negative in lesional cells. Ki-67 shows very high proliferative index. p16 is strongly positive.

2. p16 is strongly positive.

**FINAL CONCLUSION (27/06/2023):**

*Natal Cleft Right:*

- MODERATELY DIFFERENTIATED BASALOID SQUAMOUS CELL CARCINOMA (SCC)

- p16 POSITIVE

*Natal Cleft Left:*

- HYPERTROPHIC AND BASALOID INTRAEPIDERMAL SQUAMOUS CELL CARCINOMA

- p16 POSITIVE

Appendix B: Radiological Results

**MRI-Abdomen and Pelvis**

TECHNIQUE:
Multi sequence and multiplanar MRI of the pelvis performed as pre and post contrast T1, T2, and diffusion weighted imaging.

FINDINGS:
There is an area of irregular enhancement in the natal cleft measuring 6 x 13 mm in axial plane (partially imaged axially) and approximately 28 mm in craniocaudal diameter which could potentially correspond with the history of SCC although otherwise non-specific with no other visible mass lesion. There is no visible invasion of any underlying structures. A small subcutaneous cystic focus is noted measuring 5 mm to the left side of the superior margin of the natal cleft. Previous right total hip replacement. A left pelvic cyst is similar to previous studies measuring approximately 61 mm in diameter. Sacral Tarlov cysts are noted. History of a previous coccygeal resection. Marrow signal is heterogeneous but no definite metastases are identified.

CONCLUSION:
The history of a natal cleft SCC presumably corresponds with an area of irregular enhancement measuring up to 28 mm in craniocaudal diameter but appears confined to the cutaneous and superficial subcutaneous tissues. No invasion of underlying structures is demonstrated.

**CT-Chest, Abdomen and Pelvis**

TECHNIQUE:
IV contrasted CT of the chest, abdomen and pelvis with multiplanar reformations

FINDINGS:
*CT thorax:*
No mediastinal, hilar or axillary lymphadenopathy. There has been previous coronary artery bypass graft surgery. No pericardial or pleural effusion. There are several mixed density plaques noted at the anterior and anterolateral mid zones bilaterally, is there a history of prior asbestos exposure? 4.7 mm centrilobular nodule left lower lobe. Non-specific but possibly postinflammatory. This could be reviewed at 6 to 12-month follow-up. No suspicious pulmonary lesion is noted otherwise. Chronic anterior wedge compression fracture of T12 with approximately 60% loss of the anterior vertebral body height.
*CT abdomen and pelvis:*
There has been previous surgery within the upper abdomen, which looks to include splenectomy and distal pancreatectomy. Liver normal appearance. Cholelithiasis. Remaining pancreas normal. Both adrenals and kidneys normal. No abdominal or pelvic lymphadenopathy. 58 mm cyst within the pelvis on the left may be ovarian in origin. It is unchanged from the previous MRI 10th March 2023. Postsurgical/post therapy changes are noted within the coccyx and distal sacrum with minor stranding of the soft tissues. And obvious masses not identified on CT. No ascites.

CONCLUSION:
No evidence of metastatic disease. Evidence of previous distal sacral/coccygeal resection. Left adnexal cyst 58 mm diameter, possibly ovarian in origin and unchanged from prior MRI from March 2023. Pleural plaques both hemithoraces, possible previous asbestos exposure.
